# Development of a Machine Learning Model to Estimate US Firearm Homicides in Near Real Time

**DOI:** 10.1001/jamanetworkopen.2023.3413

**Published:** 2023-03-17

**Authors:** Elizabeth A. Swedo, Alen Alic, Royal K. Law, Steven A. Sumner, May S. Chen, Marissa L. Zwald, Miriam E. Van Dyke, Daniel A. Bowen, James A. Mercy

**Affiliations:** 1 Division of Violence Prevention, National Center for Injury Prevention and Control, Centers for Disease Control and Prevention, Atlanta, Georgia; 2 Division of Injury Prevention, National Center for Injury Prevention and Control, Centers for Disease Control and Prevention, Atlanta, Georgia; 3 Office of Strategy and Innovation, National Center for Injury Prevention and Control, Centers for Disease Control and Prevention, Atlanta, Georgia; 4 Epidemic Intelligence Service, Center for Surveillance, Epidemiology, and Laboratory Services, Centers for Disease Control and Prevention, Atlanta, Georgia

## Abstract

**Importance:**

Firearm homicides are a major public health concern; lack of timely mortality data presents considerable challenges to effective response. Near real-time data sources offer potential for more timely estimation of firearm homicides.

**Objective:**

To estimate near real-time burden of weekly and annual firearm homicides in the US.

**Design, Setting, and Participants:**

In this prognostic study, anonymous, longitudinal time series data were obtained from multiple data sources, including Google and YouTube search trends related to firearms (2014-2019), emergency department visits for firearm injuries (National Syndromic Surveillance Program, 2014-2019), emergency medical service activations for firearm-related injuries (biospatial, 2014-2019), and National Domestic Violence Hotline contacts flagged with the keyword *firearm* (2016-2019). Data analysis was performed from September 2021 to September 2022.

**Main Outcomes and Measures:**

Weekly estimates of US firearm homicides were calculated using a 2-phase pipeline, first fitting optimal machine learning models for each data stream and then combining the best individual models into a stacked ensemble model. Model accuracy was assessed by comparing predictions of firearm homicides in 2019 to actual firearm homicides identified by National Vital Statistics System death certificates. Results were also compared with a SARIMA (seasonal autoregressive integrated moving average) model, a common method to forecast injury mortality.

**Results:**

Both individual and ensemble models yielded highly accurate estimates of firearm homicides. Individual models’ mean error for weekly estimates of firearm homicides (root mean square error) varied from 24.95 for emergency department visits to 31.29 for SARIMA forecasting. Ensemble models combining data sources had lower weekly mean error and higher annual accuracy than individual data sources: the all-source ensemble model had a weekly root mean square error of 24.46 deaths and full-year accuracy of 99.74%, predicting the total number of firearm homicides in 2019 within 38 deaths for the entire year (compared with 95.48% accuracy and 652 deaths for the SARIMA model). The model decreased the time lag of reporting weekly firearm homicides from 7 to 8 months to approximately 6 weeks.

**Conclusions and Relevance:**

In this prognostic study of diverse secondary data on machine learning, ensemble modeling produced accurate near real-time estimates of weekly and annual firearm homicides and substantially decreased data source time lags. Ensemble model forecasts can accelerate public health practitioners’ and policy makers’ ability to respond to unanticipated shifts in firearm homicides.

## Key Points

**Question:**

Can diverse secondary data sources accurately estimate US firearm homicides in near real time?

**Findings:**

This national prognostic study combines data from 5 online, health service, and hotline data sources into an ensemble model that accurately (99.74%) forecasted firearm homicide deaths in near real time within 38 deaths for the year.

**Meaning:**

The findings of this study suggest that this model for forecasting firearm homicides provides a viable process to facilitate timely prevention efforts and expand firearm violence research using secondary data sources.

## Teaser

This prognostic study uses data from publicly available online, health service, and hotline data sources as inputs into machine learning models to estimate the weekly and annual burden of firearm homicides and discusses the utility of this approach for public health prevention.

## Introduction

Firearm homicides are a leading public health concern in the US. Between 2019 and 2020, US firearm homicide rates increased by 35%, whereas racial disparities in rates widened, underscoring this significant problem.^1^ Provisional vital statistics indicate that firearm homicide rates increased again in 2021 to approximately 20 966 firearm homicides—8.3% higher in 2021 than 2020.^2^ Firearm homicides are among the top 5 leading causes of death among US individuals aged 1 to 44 years.^3^ Despite the scope of this problem, it is difficult to describe and anticipate changes in national trends in firearm homicide in a timely fashion. Firearm mortality data reported by the Centers for Disease Control and Prevention (CDC) are derived from death certificates completed by more than 2000 local coroner and/or medical examiner offices nationwide.^4^ As a result of this significant decentralization in vital records, compiling provisional mortality statistics takes approximately 7 to 8 months, and final national firearm mortality statistics lag by more than 1 year.^3^ Although some local law enforcement agencies collect and publish near real-time data,^5^ the ability to examine national homicide data is significantly limited by recent diminished law enforcement participation in the Federal Bureau of Investigation’s voluntary National Incident-Based Reporting System.^6,7^

Data lags present significant challenges to public health response efforts.^8^ Delays in firearm homicide data impact federal, state, and local agencies’ ability to monitor trends, examine the problem’s magnitude, and make timely data-informed decisions.^8^ Such delays impede efforts to fund, support, and coordinate evidence-based, timely responses to prevent firearm violence.^9^ Finally, accurate and timely surveillance data are critical for identifying trends among populations that might benefit from prevention services and for allocating resources accordingly.

The availability of near real-time data provides an opportunity to advance timely estimation of firearm homicides. Secondary data sources, such as social media and search query data, have been used to track and predict various health outcomes, including mental health and suicide.^10,11,12,13,14^ Although a previous study^15^ observed a strong correlation between firearm-related online search data and firearm death rates across US states, limited work has been done to combine information from multiple data sources.

Drawing from previous successes estimating suicide and opioid overdose fatalities using machine learning approaches,^10,14^ this study uses anonymous time series count data from online, publicly available health service and domestic violence hotline data sources as inputs into machine learning models to estimate the weekly and annual burden of firearm homicides in near real time and for forecasted periods and discusses the utility of this approach for public health prevention.

## Methods

For this prognostic study, we used anonymous time series count data from 5 online and health service predictor data sources and 1 outcome data source to build the model. This activity was reviewed by the CDC and conducted consistent with applicable federal law and CDC policy (45 CFR §46, 21 CFR §56, 42 USC §241(d), 5 USC §552a, and 44 USC §3501 et seq); as a secondary analysis of deidentified data, the study was exempt from CDC institutional review board review and the requirement for informed consent. This study adhered to Strengthening the Reporting of Observational Studies in Epidemiology (STROBE) and Transparent Reporting of a Multivariable Prediction Model for Individual Prognosis or Diagnosis (TRIPOD) reporting guidelines. No informed consent was required.

### Data Sources

All data were publicly available and/or deidentified, theoretically associated with firearm homicide trends, and (apart from death certificate data) available in real time or near real time. Online data sources were included in the model as proxies for public interest in firearms. Health service data sources were included to reflect care-seeking for serious injuries related to firearms. National Domestic Violence Hotline data captured concerns of escalating domestic violence as a marker of potential firearm homicides by an intimate partner.

#### Death Certificate Data 

Our primary outcome of interest was weekly counts of US firearm homicide deaths. Deaths were identified from National Vital Statistics System death certificate data using *International Statistical Classification of Diseases and Related Health Problems, Tenth Revision* (*ICD-10*) underlying cause of death codes X93 to X95 and U01.4.^16^ Daily counts of national firearm homicide deaths from January 1, 2014, to December 31, 2019, were aggregated at the weekly level.

#### Online Search Trends Data

Google search term data were extracted via the Google Trends Platform using Python’s pytrends package, version 4.7^17^ to collect normalized search popularity metrics for select keywords. Ten keywords were chosen based on a review of research articles related to firearm injury and death: *gun, shotgun, rifle, 9mm, pistol, homicide, murder, gun shot, gun violence,* and *shooting.*^15^ Daily normalized counts were aggregated into continuous weekly time frames for January 1, 2014, to December 31, 2019.

#### Video Sharing Platform Trends Data

YouTube search trends were also extracted via the Google Trends Platform using Python’s pytrends package, version 4.7^17^ to collect normalized search popularity metrics for the same 10 keywords. Daily normalized counts were aggregated into a continuous weekly time frame for January 1, 2014, to December 31, 2019.

#### National Syndromic Surveillance Program 

The National Syndromic Surveillance Program (NSSP) is a collaboration among the CDC; federal, local, and state health departments; and academic and private sector partners to collect, analyze, and share electronic patient encounter data received from medical facilities, including emergency departments (EDs) in all 50 US states and Guam.^18^ The weekly proportion of ED visits related to firearm injuries from January 1, 2014, to December 31, 2019, was obtained using a validated firearm injury syndrome definition (eAppendix 1 in Supplement 1).^19^ Analyses were restricted to facilities consistently reporting informative data during the study period based on a coefficient of variation of 40 or less and mean weekly informative discharge diagnoses that were 75% complete or more.

Mean weekly informative discharge diagnosis is a measure of how informative the information in the discharge diagnosis fields is over time. The informative discharge diagnosis is used to control for the quality of the discharge diagnosis field. Facilities were included in the analysis if they had an informative discharge diagnosis greater than or equal to 75% during the past 2 years. Data quality coefficient of variation is a measure of total volume volatility over time. The coefficient of variation is used to control for onboarding of new facilities to the NSSP during the period of interest. Facilities were included if they had a coefficient of variation less than or equal to 40 during the past 2 years.

#### Near Real-time Data

The service biospatial aggregates near real-time electronic emergency medical service (EMS) patient data from a network of thousands of EMS operations in more than 40 US states^20^ and provides analytic data products comprising EMS and other electronic health care data sources for public sector and commercial health care entities. The weekly proportions of EMS activations related to firearm injuries from January 1, 2014, to December 31, 2019, were obtained using the firearm injury syndrome definition (eAppendix 2 in Supplement 1).

#### The Hotline (National Domestic Violence Hotline)

The Hotline is a 24-hour national service for survivors of domestic violence and their friends and family available via telephone, chat, and text.^21^ The Hotline records deidentified demographic and situational details of contacts; contacts tagged with the keyword *firearm* indicate that during the abuse, the perpetrator has or has had access to firearms, has threatened the use of firearms, or the individual believes their perpetrator may obtain a firearm. Call volumes to The Hotline tagged with *firearm* were extracted from January 1, 2016, to December 31, 2019, and aggregated into a continuous weekly time frame.

### Statistical Analysis

To make national, weekly predictions of firearm homicides, we implemented a multistep analytic pipeline consisting of feature selection from each data source, construction of machine learning–based predictions from each individual data source, and ensembling these predictions via a second machine learning model to yield a single composite weekly prediction. The eFigure in Supplement 1 provides a visual summary of the multistage machine learning pipeline. Consistent with contemporary machine learning practices, we divided our data into separate training, validation, and testing segments for rigorous assessment of model performance. Data from 2017 and earlier were used in model training. Data from 2018 were used in model validation (selection of the best-performing approach). Data from 2019 served as a held-out test set to assess final model performance. A baseline seasonal autoregressive integrated moving average (SARIMA) time series forecasting model, one of the most widely used approaches in injury fatality modeling,^22^ was created using mortality counts from data years before 2018 as a comparator. Analyses were conducted between September 2021 and September 2022 in R version 4.0.2 (R Foundation) and Python version 3.8.5 (Python Software Foundation).

#### Variable Selection

Variable selection procedures were used for each data source containing multiple variables to ensure that only significant features were incorporated. Six variable selection methods were tested: forward, backward, stepwise, least absolute shrinkage and selection operator (LASSO), elastic net, and random forest.^23^

#### Machine Learning Models

For individual modeling, 3 machine learning models were used to create predictions of weekly firearm homicide deaths from each single data source: linear (multivariable) regression, support vector regression, and random forests. Predictions from the best models for each individual data source were combined into a stacked ensemble model. Ensemble models were created for all data sources, health service data sources only, and online data sources only. Ensemble models tested included gradient boosting machine, neural network, generalized linear model, support vector machine, random forest, tuned random forest, and LASSO.

#### Model Performance

Three main criteria were used to assess individual and ensemble model performance. Root mean square error (RMSE) and Pearson correlation coefficient measured performance of weekly estimates. Root mean square error provides a measure of distance between predictions and actual values: the lower the RMSE, the better the model fit. Pearson correlation coefficient provides a measure of linear correlation between predictions and observed values. Full-year accuracy was assessed using the following formula:



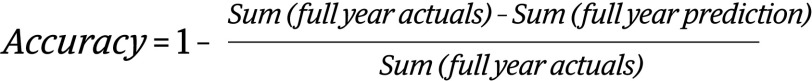



This formula provides a percentage estimate of how closely the predicted yearly death count matched the actual yearly death count. The best modeling approach was chosen using the validation data set and by averaging its position across all performance metrics. Machine learning–forecasted 2019 firearm mortality counts were compared with SARIMA model forecasts. The model decreased the lag time of rolling weekly firearm homicide estimates from 7 to 8 months (provisional death certificate data) to approximately 6 weeks (predicted data).

## Results

Table 1 details the performance of each model predicting weekly firearm homicide counts in 2019. Pearson correlation coefficients were highest for the 2 health service data sources—ED visits (*r* = 0.6502) and EMS activations (*r* = 0.6593). Pearson correlation coefficients were moderate for online data sources (*r* = 0.4551-0.4895) and low for telephone hotline data (*r* = 0.1263). Most online and health service data sources had similar mean error for weekly predictions (RMSE values); ED data performed best on this metric (RMSE = 24.95). For full-year annual prediction accuracy, online data sources predicted the number of firearm homicide deaths with greater than 99% accuracy, whereas ED data sources had 96.87% accuracy and EMS data sources had 97.32% accuracy. The domestic abuse hotline data had 98.19% full-year accuracy. The SARIMA model performed comparatively lower on most performance metrics (full-year accuracy, 95.48%; *r* = 0.4054; RMSE = 31.29).

**Table 1.** Performance of Individual Firearm Homicide Estimation Models on 2019 Testing Data Set, Built Using Each Data Source at the Intermediate Stage^a^

Data source	Model	Full-year accuracy, %	Actual deaths minus predicted deaths^b^	Pearson correlation coefficient	Root mean square error
Death certificates	SARIMA forecast	95.48	652	0.4054	31.29
**Health services**					
Emergency department visits	Multivariate linear regression	96.87	451	0.6502	24.95
Emergency medical service visits	Multivariate linear regression	97.32	386	0.6593	26.05
**Online**					
Search engine	Multivariate linear regression	99.35	94	0.4895	28.17
Vide-sharing platform	Support vector machine	99.88	18	0.4551	27.33
Domestic violence hotline	Support vector machine	98.19	261	0.1263	30.86

Abbreviation: SARIMA, seasonal autoregressive integrated moving average.

^a^ Intermediate stage represents building and evaluating models based only on each individual data source before ensembling all data sources into the final model.

^b^ Rounded to nearest whole number.

The metrics in Table 1 indicate the prediction performance on the 2019 test data set using only a single data source at a time and applying the best model and variable selection procedures identified from the testing and validation data sets. Linear models proved to be the best performers for Google, EMS, and ED data; support vector machine models performed best for YouTube and the domestic abuse hotline data.

**Figure 1.**
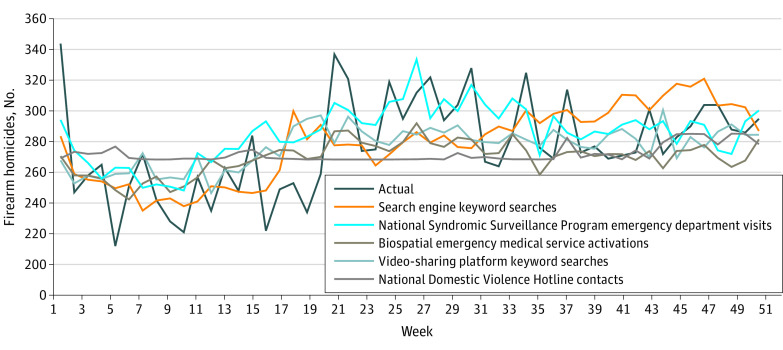
Actual and Predicted Weekly Number of Firearm Homicides in 2019 According to Individual Data Sources Individual prediction models for 2019 firearm homicide deaths were built using 5 individual data sources: search engine and video-sharing platform search trends related to firearms (2014-2019), emergency department visits for firearm injuries (National Syndromic Surveillance Program, 2014-2019), emergency medical service activations for firearm-related injuries (biospatial, 2014-2019), and domestic violence hotline contacts flagged with the keyword *firearm* (2016-2019).

Figure 1 visualizes predicted weekly deaths from individual data source models against actual weekly firearm homicide deaths. Predicted trends from individual data sources differed considerably from one another, with some overestimating or underestimating firearm homicide deaths in each week. Figure 1 demonstrates the potential unique and complementary contributions of individual data sources to an ensemble model. For example, only the NSSP adequately forecasted the 2019 summer uptick (weeks 21-31). Even data sources with generally poor predictive performance metrics, such as the domestic abuse hotline, offered helpful input at various points in the year: the predictive model built using the hotline data did not mirror actual data until week 42. Final ensemble models were built with and without the domestic abuse hotline data; inclusion of these data resulted in slightly increased performance (RMSE = 24.46 vs 24.54).

Table 2 summarizes performance metrics for the ensemble models. The ensemble model with all data sources had the highest full-year accuracy (99.74%) and a strong RMSE (24.46). The health services data ensemble model had a slightly higher Pearson correlation coefficient (*r* = 0.6728) than all data sources (*r* = 0.6036) and online data sources (*r* = 0.4621). All ensemble models combined predictions via LASSO because it consistently performed best among the multiple models evaluated. The eTable in Supplement 1 details the performance of the various machine learning models tested for the stacked ensemble model.

**Table 2.** Performance of Ensemble Firearm Homicide Estimation Models on 2019 Testing Data Set, Built Using Different Combinations of the Data Sources

Ensemble type	Model	Full-year accuracy, %	Actual deaths minus predicted deaths^a^	Pearson correlation coefficient	Root mean square error
Baseline	SARIMA	95.48	652	0.4054	31.29
Health services data sources^b^	LASSO	99.14	125	0.6728	23.01
Online data sources^c^	LASSO	98.63	197	0.4621	27.52
All data sources	LASSO	99.74	38	0.6036	24.46

Abbreviations: LASSO, least absolute shrinkage and selection operator; SARIMA, seasonal autoregressive integrated moving average.

^a^ Rounded to nearest whole number.

^b^ Health services data sources include emergency department visits and emergency medical services.

^c^ Online data sources include a search engine, video-sharing platform, and domestic violence hotline.

**Figure 2.**
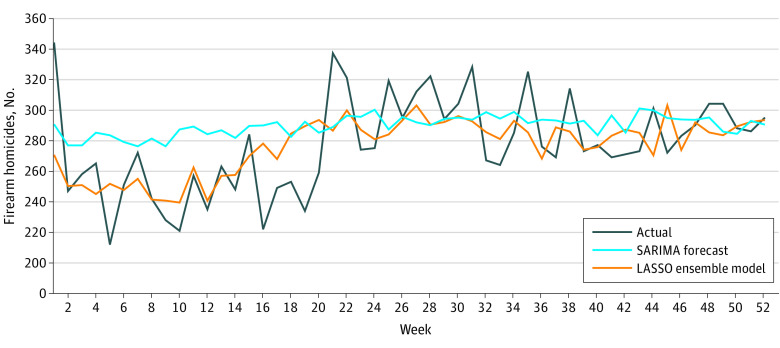
Actual and Predicted Weekly Number of Firearm Homicides in 2019 According to the Ensemble Model Final least absolute shrinkage and selection operator (LASSO) ensemble model combines all data sources (emergency department visits, emergency medical service activations, search engine and video-sharing platform keyword search, and domestic violence hotline calls) trained on 2016-2017 data, validated on 2018 data, and tested on 2019 data to predict weekly firearm homicides using a LASSO ensemble model compared with the actual number of weekly firearm homicides and seasonal autoregressive integrated moving average (SARIMA)–forecasted number of weekly firearm homicides in 2019.

Figure 2 plots predicted weekly firearm homicides using the final LASSO ensemble model. Predictions from the ensemble model followed actual firearm homicide trends and seasonality quite closely. The ensemble model failed to mirror the decrease in firearm homicides in weeks 16 to 20 because none of the individual data sources predicted the decrease. Notably, all combinations of ensemble models performed better than the baseline SARIMA model for all metrics.

## Discussion

Providing timely data is critical for firearm injury prevention efforts. We present a novel way to gather near real-time, accurate information on national firearm homicides. By combining information from online sources, emergency services, and hotline contacts, we predicted annual firearm homicide deaths with a high degree of accuracy, within 38 deaths. The current data lag between death and provisional vital statistics reporting is approximately 8 months; with ensemble methods, we accurately estimate firearm homicides in a rolling fashion with a lag time of only 6 weeks. This method could substantially decrease the impact of traditional data source time lags and accelerate multiple sectors’ ability to respond to unanticipated shifts in firearm homicides.

Timely data are necessary to make the best possible decisions. Leaders worldwide use near real-time COVID-19 data on infections, hospitalizations, and deaths to guide decisions about public health and safety^24,25^; states use near real-time syndromic surveillance data to detect, understand, and monitor disease outbreaks and bioterrorist threats.^18^ Ensemble modeling accelerates data availability, improves accuracy, and turns varied, novel data sources—such as online search trends and EMS activations for firearm injuries—into useful, actionable information to inform timely public health response to gun violence. Our methods complement case-level data compiled by Gun Violence Archive,^26^ presenting macrolevel national epidemiologic trends. Bringing together varied data sources has proven to be a successful method for violence prevention: the Cardiff Model, first fully implemented in 2001 in Cardiff, Wales, demonstrated that violence is more effectively reduced if prevention is based on information collected in both emergency departments and police departments.^27^ Ensemble modeling embodies this public health concept that many data sources are often necessary to reveal the true picture of what is happening. By combining predictions from individual data sources, the ensemble model predicted firearm homicides with better accuracy than individual data sources.

Although machine learning models are increasingly being used to forecast and study infectious diseases (eg, influenza-like illness^28^ and COVID-19^29^), applying these methods to noninfectious diseases is recent.^10,14^ Reporting of infectious diseases is much faster than for noninfectious diseases (eg, 1-2 weeks for influenza^30^ vs 1 year for topics such as suicide and firearm injury); as a result, infectious disease models use the previous week’s standard data (eg, infection rates, hospitalizations, and deaths) to build timely forecasts. For noninfectious events, such as suicide and homicide, such data (eg, deaths) are not available for up to 1 year. Combining varied, nontraditional, near real-time data sources is emerging as a viable method for forecasting noninfectious deaths. In 2020, Choi et al^10^ used a robust quantitative approach to demonstrate that novel data sources and ensemble modeling can accurately estimate near real-time suicide fatalities. With our ensemble model, we prove that these methods can be replicated for firearm homicides. Accurate short-term forecasting of infectious diseases allows for improved allocation of resources, such as hospital beds and vaccines; similarly, near real-time estimates of injury and violence can optimize the federal public health response to these problems. More timely data on firearm homicides could inform the timing and scale of implementing comprehensive prevention approaches to preventing firearm violence by providing early warnings to help guide federal planning and funding. Near real-time data also provide a timelier feedback loop by which effectiveness of efforts can be more readily assessed.

Our methods also reinforce the importance of using varied metrics to evaluate machine learning model performance. In our study, the ED-based individual model had the highest Pearson correlation coefficient; however, if we had selected our model based on the correlation coefficient alone, we would have chosen the least accurate model.

The success of this method for estimating firearm homicides represents an opportunity to accelerate prevention efforts for the multiple sectors invested in understanding firearm violence–related trends nationwide. Another crucial next step is identifying an optimal way to rapidly disseminate such data so practitioners can quickly respond to emerging trends; dissemination avenues could include data dashboards, websites like COVID-19 Data Tracker or FluView,^25,30^ or alerts disseminated through existing surveillance systems. Other potential next steps include expansion of these methods to other firearm-related causes of death, such as unintentional firearm deaths, and other injury topics. Future work is needed to explore the feasibility of estimating firearm deaths at smaller geographic units and among sociodemographic categories using this method. Researching the utility of other data sources, such as news reports, social media, and information collated by the Gun Violence Archive,^26^ would also be beneficial.

### Limitations

Although this method is promising for its timeliness and accuracy, there are limitations. First, we present national estimates; it is unclear if this modeling approach will work for smaller geographic units. Because many effective homicide prevention efforts take place at the local level, such as the Cure Violence model of local “violence interrupters” to address and deescalate disputes,^31^ having estimates at the city, county, and state levels would be beneficial. Similarly, this method does not provide estimates broken down by sociodemographic categories. Firearm homicides disproportionately impact racial and ethnic minority youth^32^; where possible, disaggregating data streams by age and race to calculate stratified estimates would provide valuable, timely information for interventions to reduce disparities. It is unknown how this method will perform in the setting of abrupt spikes, such as mass shootings and COVID-19–related increases in violence in 2020 to 2021.^33^ Further investigation with additional years of data is merited as death certificate data are released. Also, it is unknown how public health and other sectors will react to predicted data; these models are only valuable if they are used to enact more timely change. Despite the model’s accuracy, the relative newness of this method may limit actionability.

## Conclusions

In this prognostic study of diverse secondary data on machine learning, ensemble modeling produced accurate, near real-time estimates of weekly and annual firearm homicides and substantially decreased data source time lags. Ensemble model forecasts may accelerate public health practitioners’ and policy makers’ ability to respond to unanticipated shifts in firearm homicides.

## Notes

Supplement 1. **eAppendix 1.** NSSP Syndrome Definition **eAppendix 2.** biospatial Firearm Syndrome Definition **eTable.** Performance of Various Ensemble Firearm Homicide Estimation Models Considered on 2018 Validation Data Set **eFigure.** Framework and Data Sources for the Multistage Pipeline to Estimate Firearm Homicide Fatalities

Supplement 2.
Data Sharing Statement
