# Supplementary material for: Development of a Machine Learning Model to Estimate US Firearm Homicides in Near Real Time
Source: JAMA Netw Open. 2023 Mar 17;6(3):e233413. doi: 10.1001/jamanetworkopen.2023.3413 (PMC10024196; doi:10.1001/jamanetworkopen.2023.3413)
Supplement: Supplement 1. — eAppendix 1. NSSP Syndrome Definition eAppendix 2. biospatial Firearm Syndrome Definition eTable. Performance of Various Ensemble Firearm Homicide Estimation Models Considered on 2018 Validation Data Set eFigure. Framework and Data Sources for the Multistage Pipeline to Estimate Firearm Homicide Fatalities [file jamanetwopen-e233413-s001.pdf]

Supplementary Online Content

Swedo EA, Alic A, Law RK, et al. Development of a machine learning model to estimate US firearm homicides in near real time. *JAMA Netw Open*. 2023;6(3):e233413. doi:10.1001/jamanetworkopen.2023.3413

eAppendix 1. NSSP Syndrome Definition
eAppendix 2. biospatial Firearm Syndrome Definition
eTable. Performance of Various Ensemble Firearm Homicide Estimation Models Considered on 2018 Validation Data Set
eFigure. Framework and Data Sources for the Multistage Pipeline to Estimate Firearm Homicide Fatalities

This supplementary material has been provided by the authors to give readers additional information about their work.

© 2023 Swedo EA et al. *JAMA Network Open.*

eAppendix 1. NSSP Syndrome Definition

Syndrome definition description and chief complaint search terms, diagnosis codes, and negations included in syndrome definition for emergency department visits for firearm injuries

**Syndrome Definition (Outcome)**	Firearm Injury[a]
**Description of Syndrome Definition**	The syndromic surveillance definition to detect initial visits for a firearm injury uses both discharge diagnosis codes and chief complaint free text with exclusions. If discharge diagnosis codes indicate a firearm injury occurred, the emergency department visit is automatically included in the syndrome. If there is no discharge diagnosis code, the visit is only included if the chief complaint text has a firearm injury-related term. In addition, the exclusions are only applied to the chief complaint text when a discharge diagnosis code is not present.
**Diagnosis Codes[b]**	
International Classification of Diseases, Ninth Revision, Clinical Modification (ICD-9-CM) codes	E922(.0-.3, .8-.9) Accident cause by firearm<br>E955(.0-.4, .9) Suicide and self-inflicted injury by firearms<br>E965(.0-.4) Assault by firearms<br>E970 Injury due to legal intervention by firearms<br>E979.4 Terrorism involving firearms<br>E985(.0-.4) Injury by firearms, undetermined intent
International Classification of Diseases, Tenth Revision, Clinical Modification (ICD-10-CM) codes	W32 Accidental handgun discharge and malfunction<br>- W32.0 Accidental handgun discharge<br>- W32.1 Accidental handgun malfunction<br><br>W33 Accidental rifle, shotgun and larger firearm discharge and malfunction<br>W33.0 Accidental rifle, shotgun and larger firearm discharge<br>- W33.00 Accidental discharge of unspecified larger firearm<br>- W33.01 Accidental discharge of shotgun<br>- W33.02 Accidental discharge of hunting rifle<br>- W33.03 Accidental discharge of machine gun<br>- W33.09 Accidental discharge of other larger firearm<br><br>W33.1 Accidental rifle, shotgun and larger firearm malfunction<br>- W33.10 Accidental malfunction of unspecified larger firearm<br>- W33.11 Accidental malfunction of shotgun<br>- W33.12 Accidental malfunction of hunting rifle<br>- W33.13 Accidental malfunction of machine gun<br>- W33.19 Accidental malfunction of other larger firearm<br><br>W34 Accidental discharge and malfunction from other and unspecified firearms and guns<br>- W34.0 Accidental discharge from other and unspecified firearms and guns<br>- W34.00 Accidental discharge from unspecified firearms or gun<br>- W34.09 Accidental discharge from other specified firearms<br><br>W34.1 Accidental malfunction from other and unspecified firearms and guns<br>- W34.10 Accidental malfunction from unspecified firearms or gun<br>- W34.19 Accidental malfunction from other specified firearms<br><br>X72 Intentional self-harm by handgun discharge<br><br>X73 Intentional self-harm by rifle, shotgun and larger firearm discharge<br>- X73.0 Intentional self-harm by shotgun discharge<br>- X73.1 Intentional self-harm by hunting rifle discharge<br>- X73.2 Intentional self-harm by machine gun discharge<br>- X73.8 Intentional self-harm by other larger firearm discharge<br>- X73.9 Intentional self-harm by unspecified larger firearm discharge<br><br>X74 Intentional self-harm by other and unspecified firearm and gun discharge<br>- X74.8 Intentional self-harm by other firearm discharge<br>- X74.9 Intentional self-harm by unspecified firearm discharge<br><br>X93 Assault by handgun discharge<br><br>X94 Assault by rifle, shotgun and larger firearm discharge<br>- X94.0 Assault by shotgun<br>- X94.1 Assault by hunting rifle<br>- X94.2 Assault by machine gun<br>- X94.8 Assault by other larger firearm discharge<br>- X94.9 Assault by unspecified larger firearm discharge

© 2023 Swedo EA et al. *JAMA Network Open.*

	X95 Assault by other and unspecified firearm and gun discharge<br>- X95.8 Assault by other firearm discharge<br>- X95.9 Assault by unspecified firearm discharge<br><br>Y22 Handgun discharge, undetermined intent<br><br>Y23 Rifle, shotgun and larger firearm discharge, undetermined intent<br>- Y23.0 Shotgun discharge, undetermined intent<br>- Y23.1 Hunting rifle discharge, undetermined intent<br>- Y23.2 Military firearm discharge, undetermined intent<br>- Y23.3 Machine gun discharge, undetermined intent<br>- Y23.8 Other larger firearm discharge, undetermined intent<br>- Y23.9 Unspecified larger firearm discharge, undetermined intent<br><br>Y24 Other and unspecified firearm discharge, undetermined intent<br>- Y24.8 Other firearm discharge, undetermined intent<br>- Y24.9 Unspecified firearm discharge, undetermined intent<br><br>Y35.0 Legal intervention involving firearm discharge<br>Y35.00 Legal intervention involving unspecified firearm discharge<br>- Y35.001 Legal intervention involving unspecified firearm discharge, law enforcement official injured<br>- Y35.002 Legal intervention involving unspecified firearm discharge, bystander injured<br>- Y35.003 Legal intervention involving unspecified firearm discharge, suspect injured<br><br>Y35.01 Legal intervention involving injury by machine gun<br>- Y35.011 Legal intervention involving injury by machine gun, law enforcement official injured<br>- Y35.012 Legal intervention involving injury by machine gun, bystander injured<br>- Y35.013 Legal intervention involving injury by machine gun, suspect injured<br>Y35.02 Legal intervention involving injury by handgun<br>- Y35.021 Legal intervention involving injury by handgun, law enforcement official injured<br>- Y35.022 Legal intervention involving injury by handgun, bystander injured<br>- Y35.023 Legal intervention involving injury by handgun, suspect injured<br><br>Y35.09 Legal intervention involving other firearm discharge<br>- Y35.091 Legal intervention involving other firearm discharge, law enforcement official injured<br>- Y35.092 Legal intervention involving other firearm discharge, bystander injured<br>- Y35.093 Legal intervention involving other firearm discharge, suspect injured<br><br>Y38.4 Terrorism involving firearms<br><br>Y38.4X Terrorism involving firearms<br>- Y38.4X1 Terrorism involving firearms, public safety official injured<br>- Y38.4X2 Terrorism involving firearms, civilian injured<br>- Y38.4X3 Terrorism involving firearms, terrorist injured
Systematized Nomenclature of Medicine (SNOMED) codes	41430008, 56768003, 63409001, 69861004, 77301004, 86122002, 111050005, 219257002, 283545005, 218081007, 218086002, 218082000, 218087006, 218088001, 269796009, 242869008, 219199009, 219200007, 219201006, 219204003, 219205002, 219203009, 219198001, 219142001, 219143006, 219144000, 219145004, 219146003, 287184008, 287193009
**Chief Complaint Search Terms**	GSW<br>Gunshot (and common spellings and misspellings)<br>Buckshot<br>Revolver<br>Rifle<br>Shotgun<br>Firearm<br>Pistol<br>Handgun<br>Been shot<br>I was shot<br>I got shot<br>Combination of hit, ricochet, or graze with bullet<br>Gun and wound
**Negations**	Z51.89 or Z5189

© 2023 Swedo EA et al. *JAMA Network Open.*

	Encounter for other specified after care<br>No gun<br>Remove gun<br>Heard or hearing gun<br>Hit head<br>Kickback<br>Water gun<br>Pellet<br>BB gun (and common spellings and misspellings)<br>Rubber bullet<br>Paint ball gun<br>Nerf gun<br>Air gun<br>Spring gun<br>Pistol whip<br>Nail gun<br>Staple gun<br>Stun, laser, or Taser gun<br>Struck or hit with, in combination with gun or pistol<br>Bloodshot<br>Scope<br>Fake<br>Allergy shot<br>Injection<br>Follow up<br>Chronic<br>Wound check, care, recheck, or infection<br>Drainage<br>Gswel or Gswol<br>GSW, gunshot, or bullet and history, ago, past, prior, previous, or old

[a]Emergency department visits for firearm injuries were identified by using a syndrome definition developed by CDC in partnership with state and local health departments to query National Syndromic Surveillance Program (NSSP) data. The syndrome definition used in this report can be found in ESSENCE and labeled CDC Firearm Injury v2.

[b]Also includes diagnosis codes with no period (e.g., “W340”)

© 2023 Swedo EA et al. *JAMA Network Open.*

eAppendix 2. biospatial Firearm Syndrome Definition

A National Emergency Medical Services Information System (NEMSIS) v2 or v3 record was labeled as a firearm injury if one or more of the following was true:

- Cause of Injury (E10_01) is one of the following:
    - 9560: 'Firearm Assault (E965.0)'
    - 9565: 'Firearm Injury (accidental) (E985.0)'
    - 9570: 'Firearm self inflicted (E955.0)'
- Cause of Injury (eInjury.01) indicates any of the following ICD-10 codes (sub-codes included): W32-W33, W34.00, W34.09, W34.10, W34.19, X72, X73, X74.8, X74.9, X93, X94, X95.8, X95.9, Y22, Y23, Y24.8, Y24.9, Y35.01, Y35.02, Y35.09, Y38.4.
- Chief/secondary complaint (E09_05, E09_08; eSituation.04) contains a key word or phrase related to firearm-related injury: ”gsw”, "gun shot", "shooting", “bullet graze”, or variants of “hit…bullet” and “graze…bullet”.
- **Both** of the following are true:
    - Narrative (E13_01; eNarrative.01) contains a key word or phrase related to firearm-related injury: ”gsw”, "gun shot wound", “bullet graze”, or variants of “hit…bullet” and “graze…bullet”.
    - Complaint reported by dispatch (E03_01; eDispatch.01) is '530' - "Stab/Gunshot Wound" or '2301063' - "Stab/Gunshot Wound/Penetrating Trauma".

Exclusions:

- Provider impressions (eSituation.11, eSituation.12) indicates Z51.89 “Encounter for other specified aftercare”.
- Incident/Patient Disposition (E20_10, eDisposition.12) indicates cancelled prior to arrival at scene, cancelled on scene (no patient contact or found), or standby (no services or support provided).

eTable. Performance of Various Ensemble Firearm Homicide Estimation Models Considered on 2018 Validation Data Set

Year	Model	Full Year Accuracy	|Actual Deaths – Predicted Deaths|[a]	Pearson Correlation Coefficient	Root Mean Square Error for Training Data	Root Mean Square Error for Testing Data
2018 (Validation)	Gradient Boosting Machine	96.87%	437	0.26	16.57	24.83
	Neural Network	96.35%	510	0.10	28.83	25.78
	Generalized Linear Model	98.23%	247	0.36	17.38	22.82
	Support-Vector Machine	98.47%	213	0.19	15.63	23.92
	Random Forest	98.72%	178	0.10	9.75	24.97
	Tuned Random Forest	98.46%	214	0.15	8.94	24.84
	LASSO[b]	98.75%	175	0.34	17.47	22.67

[a]Rounded to nearest whole number.

[b]Using the 2018 validation data, LASSO was chosen as the stacked ensemble model for the 2019 test data

© 2023 Swedo EA et al. *JAMA Network Open.*

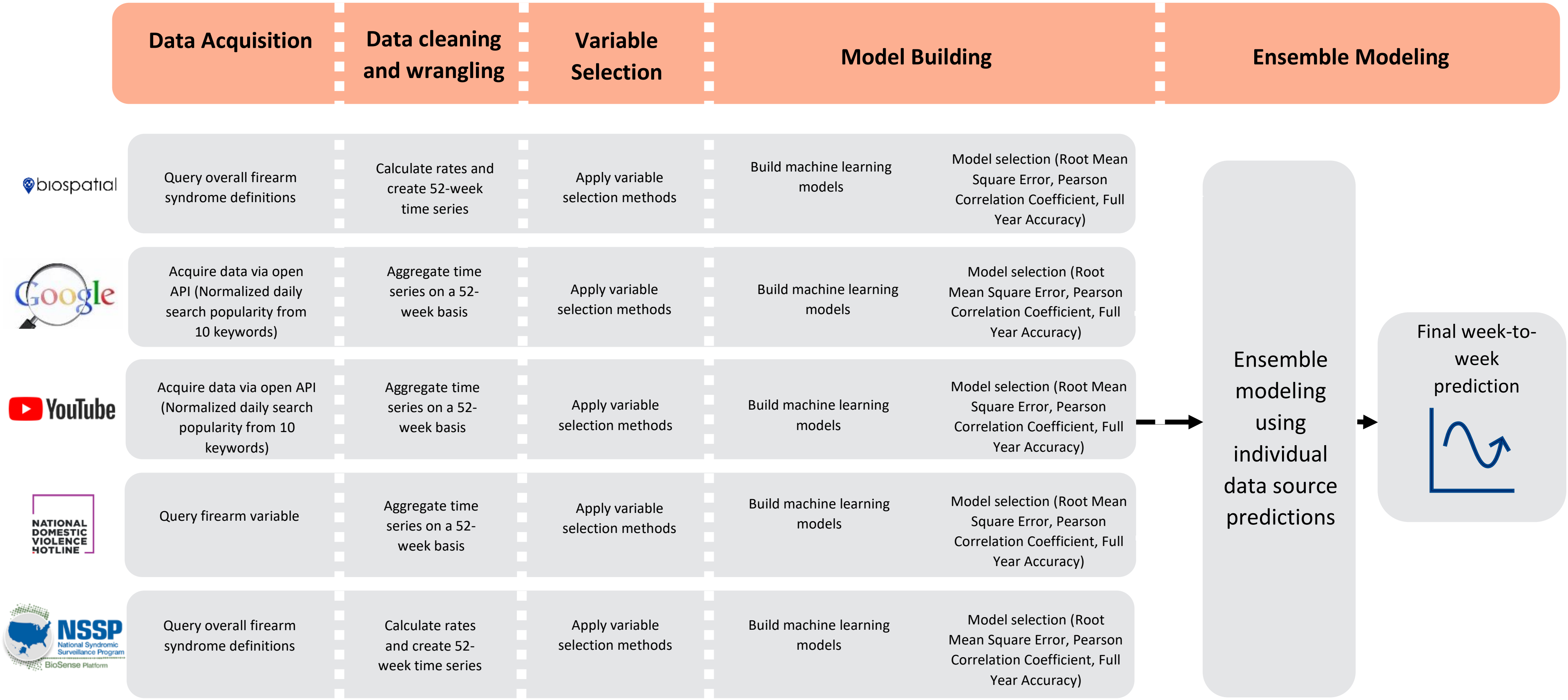

eFigure. Framework and Data Sources for the Multistage Pipeline to Estimate Firearm Homicide Fatalities

The first phases consisted of Data Acquisition and Data Preparation to allow each individual data source to be consolidated in the same manner. Variable selection was then performed for data sources with multiple variables before fitting them into individual models. Once individual models were assessed and the best model was chosen for each data source, the final step of Ensemble Modeling was performed to develop the final prediction.

© 2023 Swedo EA et al. *JAMA Network Open.*
