# Supplementary material for: Development of a Machine Learning Model to Estimate US Firearm Homicides in Near Real Time
Source: JAMA Netw Open. 2023 Mar 17;6(3):e233413. doi: 10.1001/jamanetworkopen.2023.3413 (PMC10024196; doi:10.1001/jamanetworkopen.2023.3413)
Supplement: Supplement 2. — Data Sharing Statement [file jamanetwopen-e233413-s002.pdf]

Data Sharing Statement

Swedo. Development of a Machine Learning Model to Estimate US Firearm Homicides in Near Real Time. *JAMA Netw Open*. Published March 17, 2023.
doi:10.1001/jamanetworkopen.2023.3413

Data
Data available: Yes
Data types: Other (please specify)
Additional Information: Data underlying the results reported in this Article were used under license and agreement with the respective organizations and agencies owning such data. Where existing data use agreements allow, data will be shared by the corresponding author; for other data sources, the corresponding author will connect inquirers to the appropriate entity for data access. Analytical code is available at https://github.com/qlu9/REDUFVE.
How to access data: eswedo@cdc.gov
When available: With publication

Supporting Documents
Document types: Statistical/analytic code
How to access documents: https://github.com/qlu9/REDUFVE
When available: With publication

Additional Information
Who can access the data: Anyone
Types of analyses: Any purpose
Mechanisms of data availability: Data underlying the results reported in this Article were used under license and agreement with the respective organizations and agencies owning such data. Where existing data use agreements allow, data will be shared by the corresponding author; for other data sources, the corresponding author will connect inquirers to the appropriate entity for data access.
